# Evidence for Thermodynamically Stable “On-Top” Sulfur Divacancy in Monolayer WS_2_

**DOI:** 10.1021/acsnanoscienceau.5c00165

**Published:** 2026-05-20

**Authors:** Weiru Chen, John C. Thomas, Yihuang Xiong, Zhuohang Yu, Da Zhou, Shalini Kumari, Zhongwei Dai, Joshua A. Robinson, Mauricio Terrones, Archana Raja, Sinéad M. Griffin, Alexander Weber-Bargioni, Geoffroy Hautier

**Affiliations:** † Thayer School of Engineering, 3728 Dartmouth College, Hanover, New Hampshire 03755, United States; ‡ Materials Sciences Division, 682790 Lawrence Berkeley National Laboratory, Berkeley, California 94720, United States; § Molecular Foundry, Lawrence Berkeley National Laboratory, Berkeley, California 94720, United States; ∥ Department of Materials Science and Engineering, 8082 The Pennsylvania State University, University Park, Pennsylvania 16082, United States; ⊥ Center for Two-Dimensional and Layered Materials, The Pennsylvania State University, University Park, Pennsylvania 16802, United States; # Department of Physics, The Pennsylvania State University, University Park, Pennsylvania 16802, United States; ∇ Department of Chemistry, The Pennsylvania State University, University Park, Pennsylvania 16802, United States; ○ Kavli Energy NanoScience Institute, 1438 University of California Berkeley, Berkeley, California 94720, United States; ● Department of Materials Science and NanoEngineering, 539850 Rice University, Houston, Texas 77005, United States; ▲ Rice Advanced Materials Institute, Rice University, Houston, Texas 77005, United States

**Keywords:** monolayer WS_2_, sulfur vacancy complex, defect binding energy, first-principle calculations, scanning tunneling spectroscopy

## Abstract

Chalcogen vacancies in monolayer transition metal dichalcogenides (TMDs), such as WS_2_, play a crucial role in various applications ranging from optoelectronics and catalysis to quantum information science (QIS), making their identification and control essential. This study focuses on the WS_2_ single vacancy and vacancy pairs. Using first-principles computations, we investigate their thermodynamic stabilities and electronic structures. We identify an ”on-top” divacancy configuration where two vacancies sit on top of each other to be the only energetically stable complex with a binding energy of 160 meV. We compute a small difference in electronic structure with a shift of the unoccupied state by 140 meV for the divacancy complex and make note of a similar shift observed experimentally.

## Graphical abstract

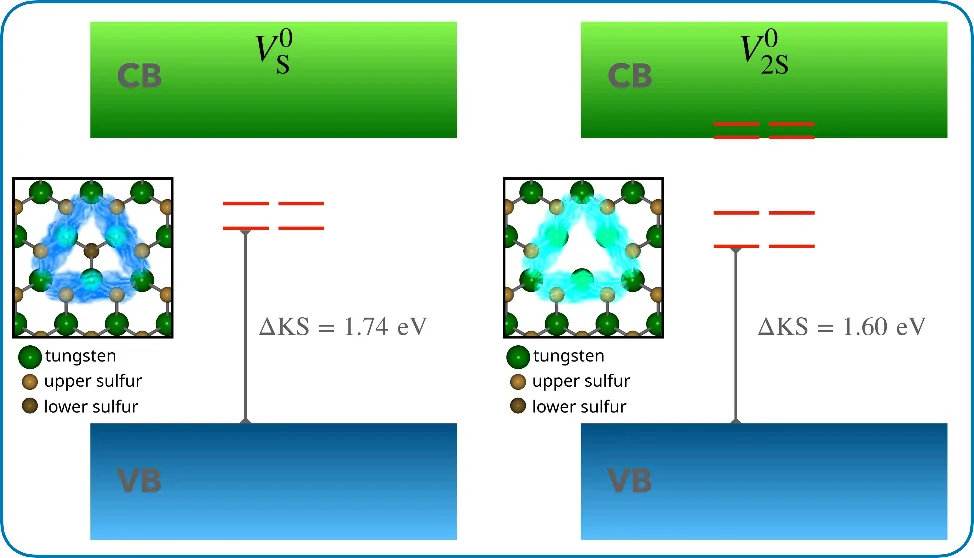

Transition metal dichalcogenides (TMDs) MX_2_ (M = transition metal atoms: W, Mo; X, chalcogen atoms: S, Se) are an interesting class of semiconducting two-dimensional (2D) materials providing a wide array of rich material properties that include optoelectronic transitions, bandgap tunability, Fermi level modification, and dopant amenability.^1–4^ As a monolayer, 1H-phase TMDs exhibit semiconducting characteristics with direct band gaps in the visible range at the *K* point of the Brillouin zone.^5–7^ Their intrinsic quantum confinement nature, reduced dielectric screening, as well as strong spin–orbit coupling (SOC) have given rise to various technologically attractive phenomenons including strong electron–hole interactions,^8,9^ catalytic activities^10,11^ and high carrier mobility.^12,13^ As many semiconductors, TMDs are sensitive to structural defects, making defect identification and subsequent control functionally relevant.^14^ In particular, chalcogen vacancies are arguably the most common defect and have been shown to influence various properties of TMDs. Chalcogen vacancies have been shown to control catalysis, carrier transport, and optical emission.^15–19^ In quantum applications, chalcogen vacancies can act as single photon emitters in TMDs,^20–23^ a key component toward quantum information science applications.^24–27^ While many earlier studies have focused on single chalcogen vacancies, there is a growing interest in understanding vacancy complexes.^28^ The proximity of individual vacancies within a complex influences the effective electronic structure through wave function overlap as one potential interaction that determines their candidacy for quantum emission or catalysis. Additionally, the geometric arrangement of vacancy complexes can create novel potential landscapes, leading to the breaking of symmetries. Understanding and controlling defect complexes provide new opportunities to tailor defect-induced functionalities of 2D semiconductors. In this paper, we study the interactions of sulfur vacancy pairs in monolayer WS_2_. By using first-principles computations, we examine the thermodynamic stability and electronic structures of sulfur vacancy pairs and identify an energetically favorable divacancy configuration in which two vacancies sit on top of each other. Experimental scanning tunneling microscopy (STM) and scanning tunneling spectroscopy (STS) suggest evidence of the formation of such divacancy complex defects.

The thermodynamic stability of these defects was computed through defect formation energy using the PBE0 hybrid functional (see the Methodology in the Supporting Information). Figure 1c shows the formation energy vs Fermi level plots for sulfur single and vacancy pairs at charge states 2–, 1–, 0, 1+ and 2+ evaluated under sulfur poor conditions. 2– and 2+ states are not stable within the bandgap. The sulfur-rich conditions can be found in Figure S1. We computed a vacancy complex binding energy for all of these pairs (see Figure S2). We observe that the divacancy configuration (with two vacancies on top of each other (see Figure 1b) exhibits a favorable binding energy *E*_*b*_ ∼ 160 meV at the neutral charge state. All other vacancy complexes have negative or significantly smaller binding energy. Figure 1d illustrates this by comparing the formation energies of the divacancy and two infinitely separated vacancies. We note that this binding energy observed for the divacancy is largely unaffected if we use a different amount of exact exchange in the hybrid functionals (Figure S2b). The value of binding energy is relatively small compared to complex defects in other semiconductors which can be a few eV.^29–31^ This low binding energy indicates that the divacancy might not be very favorable at high synthesis temperatures. They could, however, form during low temperature annealing or cooling down after synthesis. We note that kinetic effects might be important and prevent their formation, despite their favorable driving force at lower temperatures. In any case, we show that there is a thermodynamic driving force at low temperature for forming these “on-top” divacancies and that they might be difficult to avoid under certain synthesis conditions. Recent work has measured the emission properties of single vacancies and vacancy complexes in WS_2_, highlighting that only the nearest-neighbor divacancy complex provides bright and stable optical emission.^28^ We note that the favorable binding energy could make the formation of these optically unfavorable “on-top” divacancy complexes difficult to bypass and could be an important challenge to address when designing future optimal single-photon emission systems with WS_2_.

**Figure 1:**
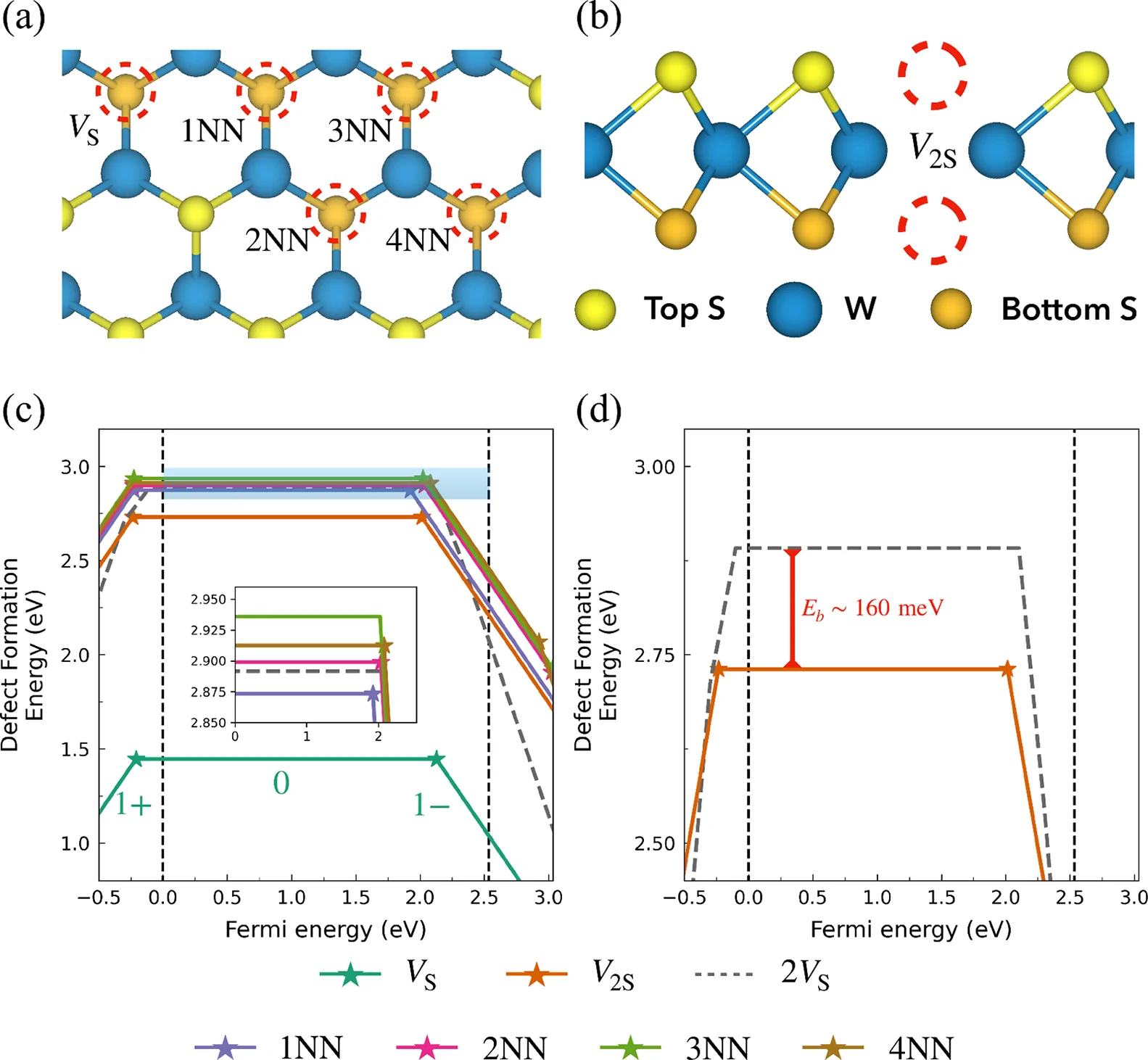
Simulated defect configurations, thermodynamic stability, and binding energy of relevant defects. (a) Sulfur single vacancy *V*_S_ and up to 4th NN vacancy complexes (*V*_S_–V_S_): 1NN, 2NN, 3NN and 4NN, separated by 3.16 Å, 5.48 Å, 6.32 Å, and 8.37 Å, respectively. (b) Sulfur divacancy configuration *V*_2S_ where two vacancies sit on top of each other. (c) Formation energy of sulfur single vacancy *V*_S_, divacancy *V*_2S_, and nearest neighbor complexes 1NN to 4NN from charge state 2– to 2+ evaluated under sulfur poor condition. Gray dashed line indicates two infinitely separated vacancies 2*V*_S_ competing with the formation of vacancy pairs. Inset shows zoomed-in energy range marked in the shaded area. (d) A binding energy around 160 meV is observed by the divacancy configuration at neutral charge state. Band edge positions are marked with vertical dashed lines. Fermi energy is referenced to the VBM.

In the electronic structure analysis of these vacancy complexes, the sulfur single vacancy possesses a *C*_3*v*_ symmetry and hosts a singlet ground state at neutral charge state with two unoccupied degenerate *e* states and a fully occupied *a*_1_ state resonant with the valence band. This *a*_1_ state can be observed clearly if we look at the 2–charge state (see Figure 2). The charge density distribution of the *e* state exhibits *d*_*x* ^2^–*y* ^2^_ and *d*_*xy*_ orbitals from the three nearby tungsten atoms while the *a*_1_ state mixes *d*_*z* ^2^_ orbitals. Similar electronic structures of single neutral chalcogen vacancy have been observed in previous studies of WS_2_ and other TMDs.^19,21,22,32–37^ We note that the band edge positions are computed using 22% Fock exchange while the defect levels are computed using 7% Fock exchange and their relative positions are corrected using both charge corrections and vacuum level alignment (see Computational Details in Supporting Information). From now on, we will only show defect states that are within the bandgap for simplicity. When we introduce one more sulfur vacancy, the electronic structures of the NN vacancy complexes converges, as expected, toward that of an isolated vacancy with increasing separation. The proximity of another vacancy splits the unoccupied levels by 0.74, 0.66, 0.18, 0.27, and 0.1 eV for the divacancy, 1NN, 2NN, 3NN, and 4NN, respectively, as shown in Figure 3a. The divacancy configuration, shown to have a favorable binding energy, exhibits a unique signature in its electronic structure. Figure 3a shows the ground state electronic structures of divacancy configuration and the wave functions associated with subsequent defect states (Figure 3b,c). The divacancy configuration has *D*_3*h*_ symmetry and hosts a singlet ground state at the neutral charge state. Interestingly, it shows two unoccupied degenerate in-gap states, akin to the single vacancy, and an additional two unoccupied degenerate states resonant with the conduction band. The wave functions of these states indicate that they are localized and originate from nearby tungsten *d* orbitals. Similar electronic structure of the divacancy configuration has been reported previously.^38^ Further incorporation of SOC splits the in-gap state degeneracy by 0.2 and 0.3 eV for the single and divacancy configuration, respectively, resulting in four doubly degenerate in-gap states for both defects. The defect states resonant with the conduction band for the divacancy configuration moves further into the conduction band (see Figure 4).

**Figure 2:**
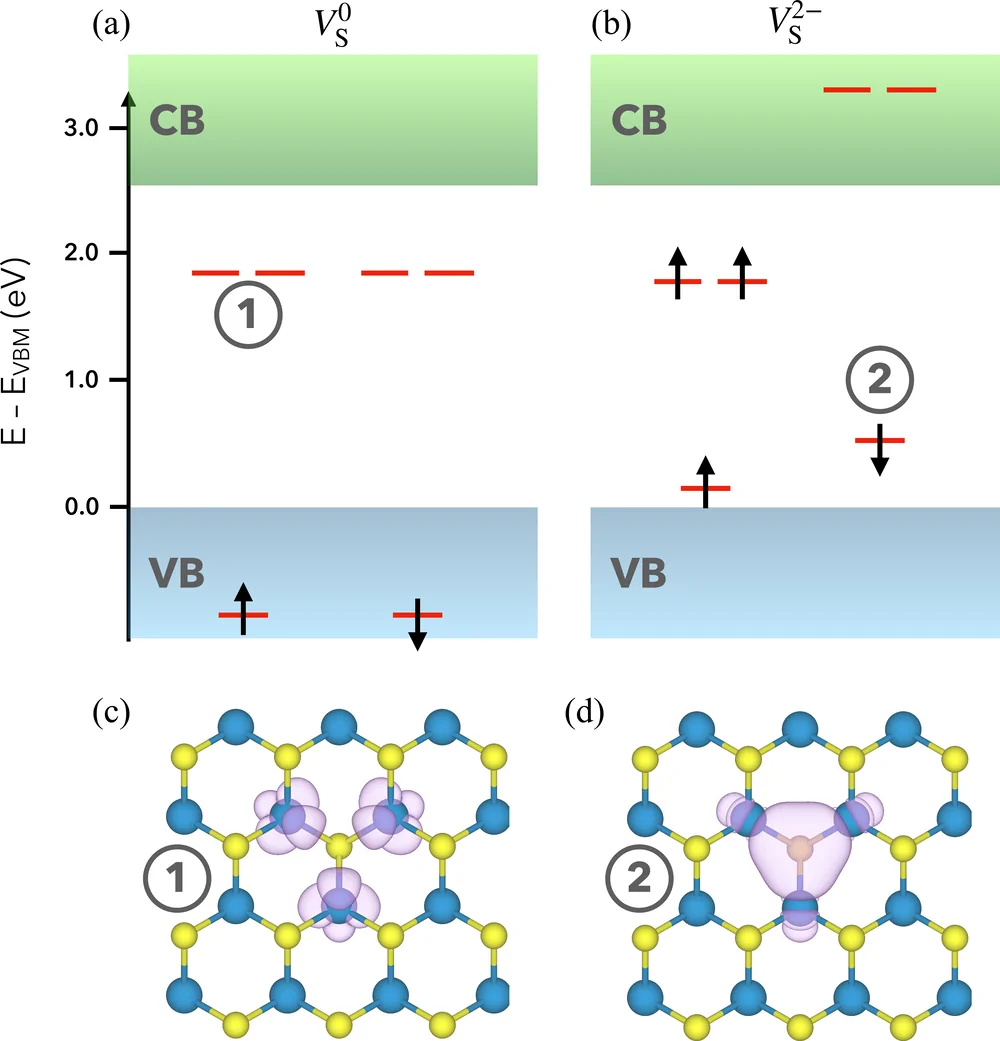
Single-particle Kohn–Sham energy levels for a single sulfur vacancy at the (a) 0 and (b) 2– state. The occupied levels at the 0 charge state are buried deep within the valence band. SOC is not applied to defect states. (c) and (d), charge density distributions for *e* and *a*_1_ states labeled in (a) and (b), respectively. Isocontour value: 0.002 Å^–3^.

**Figure 3:**
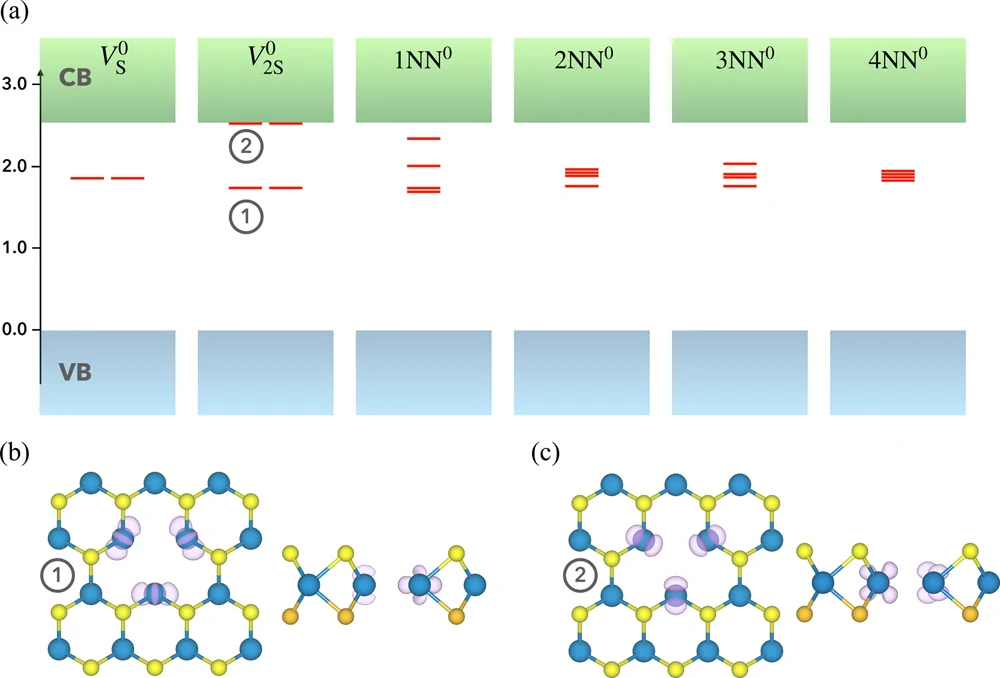
Electronic structure for sulfur single vacancy, divacancy, and nearest neighbor vacancy complexes at neutral charge state. Only one spin channel is shown for simplicity. SOC is not applied to defect levels. (b) and (c), Top and side view of the charge density distributions of the lower and higher defect states labeled by 1 and 2 in (a), respectively. Isocontour value: 0.004 Å^–3^.

**Figure 4:**
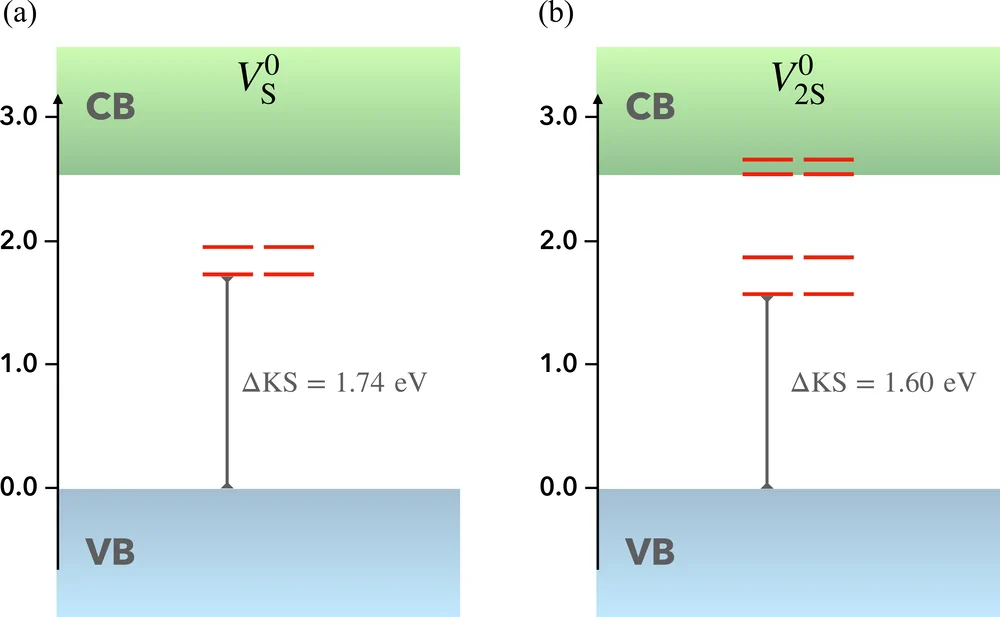
Single particle Kohn–Sham energy levels for (a) single and (b) divacancy at the neutral charge state including SOC. An electronic transition from the occupied VBM to an unoccupied defect state is described by the Kohn–Sham energy difference ΔKS.

We observe that the unoccupied defect state is shifted toward the VBM in the case of the divacancy by 140 meV with a Kohn–Sham energy difference (ΔKS) between VBM and defect state of 1.74 and 1.60 eV for the single and divacancy configuration, respectively. As photoluminescence measurements have been commonly used to study and detect chalcogen vacancies in TMDs, we can wonder if the divacancy could have a detectable optical signature. Unfortunately, currently in low temperature (77 K) photoluminescence measurements, single sulfur vacancies usually show a full width at half-maximum (FDHM) from 100 to 130 meV.^19,39^ This would make it challenging to clearly observe the divacancy signature with respect to the current sample quality. However, scanning tunneling spectroscopy (STS) can detect such a divacancy.

The computed unoccupied state shift for single and divacancy configurations suggests the possibility of detecting the divacancy signature in the experimentally acquired local density of states.^40,41^ We purposefully introduce defects in our sample through sputtering and annealing to produce a tunable amount of chalcogen vacancies.^42,43^ The sample is heated during Ar^+^ bombardment (Figure S3a), where rearrangement and formation of stable defects occur as the sample is further annealed. Two distinct V_S_ signatures can be identified with STS (Figure S3d) that are indistinguishable under conventional STM imaging, as shown in Figure S3b,c. Both signatures are characteristic of the sulfur vacancy defect with a spin–orbit split unoccupied state of 0.26 eV (Figure S3d,e). However, one of the spectrum, observed in a single defect, is downshifted by 0.16 eV from the other, where the isolated *V*_S_ electronic structure tungsten d orbital peak locations are consistent with literature values.^43^ This value is near the computed downshift of the divacancy (see Figure 4) which is around 0.14 eV. The calculated separation between states in Figure 4 shows a small increase (∼90 meV) from *V*_S_^0^ to *V*_2S_^0^ (0.22 and 0.31 eV, respectively). Acquired spectra over *V*_S_ and postulated *V*_2S_ do not exhibit this difference. CBM and VBM onsets are consistent with those of as-grown WS_2_ on 1 ML graphene, where both *V*_S_ and proposed *V*_2S_ show a charging peak as the defect shifts from a neutral charge state to an anion state with sufficient negative bias voltage. Experimental evidence suggests the local identification of a defect that points to a *V*_2S_ configuration. The authors note that only one defect is identified with STM/STS, and although tip integrity is verified on as-grown WS_2_, STS of the posited *V*_2S_ defect is reproducible, and measurement statistics of the number of *V*_S_ defects are high; additional evidence is needed to verify defect identification. The low occurrence of *V*_2S_ defects in WS_2_ has been noted in other studies.^28^ The presented data serve as the groundwork for identifying such vacancy complexes in STM/STS data. The authors anticipate that recognizing an energy shift within WS_2_ sulfur vacancy defects, as shown in Figure S3 and in Figure 4, will set the stage for additional measurement modalities, which may include differential conductance image maps at energetic defect peak locations, high resolution noncontact atomic force microscopy, and atomic force spectroscopy.^43,44^

In summary, we used first-principles computations to study the thermodynamic stability and electronic structure of sulfur divacancy and nearest neighbor vacancy complexes in monolayer WS_2_. We identified an “on-top” divacancy configuration to be the only thermodynamically stable vacancy complex with a binding energy of 160 meV. We noted a shifted electronic transition from the occupied valence band maximum to the unoccupied defect state within the band gap for the “on-top” divacancy configuration compared to that of a single vacancy. STS measurements suggest a spectroscopic signature consistent with the ”on-top” divacancy in WS_2_, however increased statistics are needed for verification. As quantum applications are considering sulfur vacancies and their related complexes for single-photon emission in WS_2_, the favorable binding energy of the “on-top” divacancy could have important implications on the generation and control of defect emitters in WS_2_.

## Supplementary Material



## Notes

First-principles computation details, formation energy for sulfur vacancy complexes, and sample preparation and measurement (PDF)
