# Supplementary material for: Evidence for Thermodynamically Stable “On-Top” Sulfur Divacancy in Monolayer WS2
Source: ACS Nanosci Au. 2026 May 20;6(4):517–22. doi: 10.1021/acsnanoscienceau.5c00165 (PMC13495497; doi:10.1021/acsnanoscienceau.5c00165)
Supplement: Supplementary file 1 [file ng5c00165_si_001.pdf]

Supporting Information for
Evidence for thermodynamically stable "on-top" sulfur divacancy in monolayer WS_2

Weiru Chen[1†], John C. Thomas[1,2,3†], Yihuang Xiong[1], Zhuohang Yu[4,5], Da Zhou[6], Shalini Kumari[4,5], Zhongwei Dai[3], Joshua A. Robinson[4,5,6,7], Mauricio Terrones[4,5,6,7], Archana Raja[3,8], Sinéad M. Griffin[2,3], Alexander Weber-Bargioni[2,3], and Geoffroy Hautier[1,9,10*]

[1]*Thayer School of Engineering, Dartmouth College, Hanover, NH 03755, United States*
[2]*Materials Sciences Division, Lawrence Berkeley National Laboratory, Berkeley, CA 94720, United States*
[3]*Molecular Foundry, Lawrence Berkeley National Laboratory, Berkeley, CA 94720, United States*
[4]*Department of Materials Science and Engineering, The Pennsylvania State University, University Park, PA 16082, United States*
[5]*Center for Two-Dimensional and Layered Materials, The Pennsylvania State University, University Park, PA, 16802, United States*
[6]*Department of Physics, The Pennsylvania State University, University Park, PA, 16802, United States*
[7]*Department of Chemistry, The Pennsylvania State University, University Park, PA, 16802, United States*
[8]*Kavli Energy NanoScience Institute, University of California Berkeley, Berkeley, CA 94720, United States*
[9]*Department of Materials Science and NanoEngineering, Rice University, Houston, TX, 77005, United States*
[10]*Rice Advanced Materials Institute, Rice University, Houston, TX, 77005, United States*
[*]*Email: geoffroy.hautier@rice.edu*
[†]*W.C. and J.C.T. contributed equally to this work.*

METHODOLOGY

Computational details

All computations were performed using Vienna Ab-initio Simulation Package (VASP)[1,2] and the projector-augmented wave (PAW) method[3] with the global hybrid functional PBE0[4,5]. Each charged defect was simulated at the dilute limit using a 144 atom orthorhombic monolayer supercell within a 24 Åvacuum layer. A plane-wave basis cutoff energy of 400 eV was used and the Brillouin zone was sampled using the Γ point only. Defect structure relaxations were performed at fixed volume until the force on each iron is less than 0.01 eV/Å. The formation energy of each charged defect was computed using PYCDT[6]. For a general defect X at charge state q, its formation energy is given by: $E^{\mathrm{f}}[X^q]$ is given by:

$$E^{\mathrm{f}}[X^q] = E_{\mathrm{tot}}[X^q] - E_{\mathrm{tot}}^{\mathrm{bulk}} - \sum n_i \mu_i + qE_F + E_{\mathrm{corr}}, \tag{1}$$

where the first two terms are the total energies of the supercell containing the defect and the pristine one, respectively. The third term takes care of the energy exchange with the chemical reservoirs when creating

the defect with n_i being the number of atoms with species i and corresponding chemical potential μ_i being added ($n_i > 0$) or removed ($n_i < 0$). The fourth term accounts for the electron exchange with the host material controlled by the Fermi level E_F, which is conventionally referenced to the valence band maximum (VBM). The last one is the correctional term for the finite-size effect, for which we employed methods proposed by Komsa et al.[7,8] as implemented in SLABCC[9]. We evaluated the defect formation energy under sulfur-rich conditions ($\Delta\mu_W = -2.53$ eV) and sulfur-poor conditions ($\Delta\mu_S = -1.26$ eV). The chemical potentials of each species are referenced to their elemental forms.

The band edge positions of pristine WS_2 was computed by incorporating 22% of Fock exchange with a dense k-point mesh of 12× 12 × 1. Defect levels were computed using 7% Fock exchange. This scheme to tune the amount of Fock exchange individually for band edge positions and defect levels is recently proposed for a proper description of the bandgap while reasonably fulfilling the generalized Koopmans' condition on defect levels[10]. Spin-orbit-coupling is taken into account throughout for the electronic structures unless otherwise specified. Both charge corrections and vacuum level alignment are included for single-particle Kohn-Sham energy levels.

Experimental preparation and measurement

Monolayer islands of WS_2 were grown on graphene/SiC substrates with an ambient pressure CVD approach. A graphene/SiC substrate with 10 mg of WO_3 powder on top was placed at the center of a quartz tube, and 400 mg of sulfur powder was placed upstream. The furnace was heated to 900 °C and the sulfur powder was heated to 250 °C using a heating belt during synthesis. A carrier gas for process throughput was used (Ar gas at 100 sccm) and the growth time was 60 min. The CVD grown WS_2/MLG/SiC was further annealed *in vacuo* at 400 °C for 2 hours.

WS_2 was sputtered with an argon ion gun (SPECS, IQE 11/35) that operated at 0.1 keV energy with 60° off-normal incidence at a pressure of 5×10^{-6} mbar and held at 600 °C. A measure of current (0.6×10^{-6} A) enabled the argon ion flux to be estimated at (1.5×10^{13} $\frac{ions}{cm^2 s}$), where the sample was irradiated for up to 10 seconds.

All measurements were performed with a Createc GmbH scanning probe microscope operating under ultrahigh vacuum (pressure $< 2\times10^{-10}$ mbar) at liquid helium temperatures ($T < 6$ K). Either etched tungsten or platinum iridium tips were used during acquisition. Tip apexes were further shaped by indentations onto a gold substrate for subsequent measurements taken over a defective substrate. STM images are taken in constant-current mode with a bias applied to the sample. STS measurements were recorded using a lock-in amplifier with a resonance frequency of 683 Hz and a modulation amplitude of 5 mV.

FIGURE S1-S3

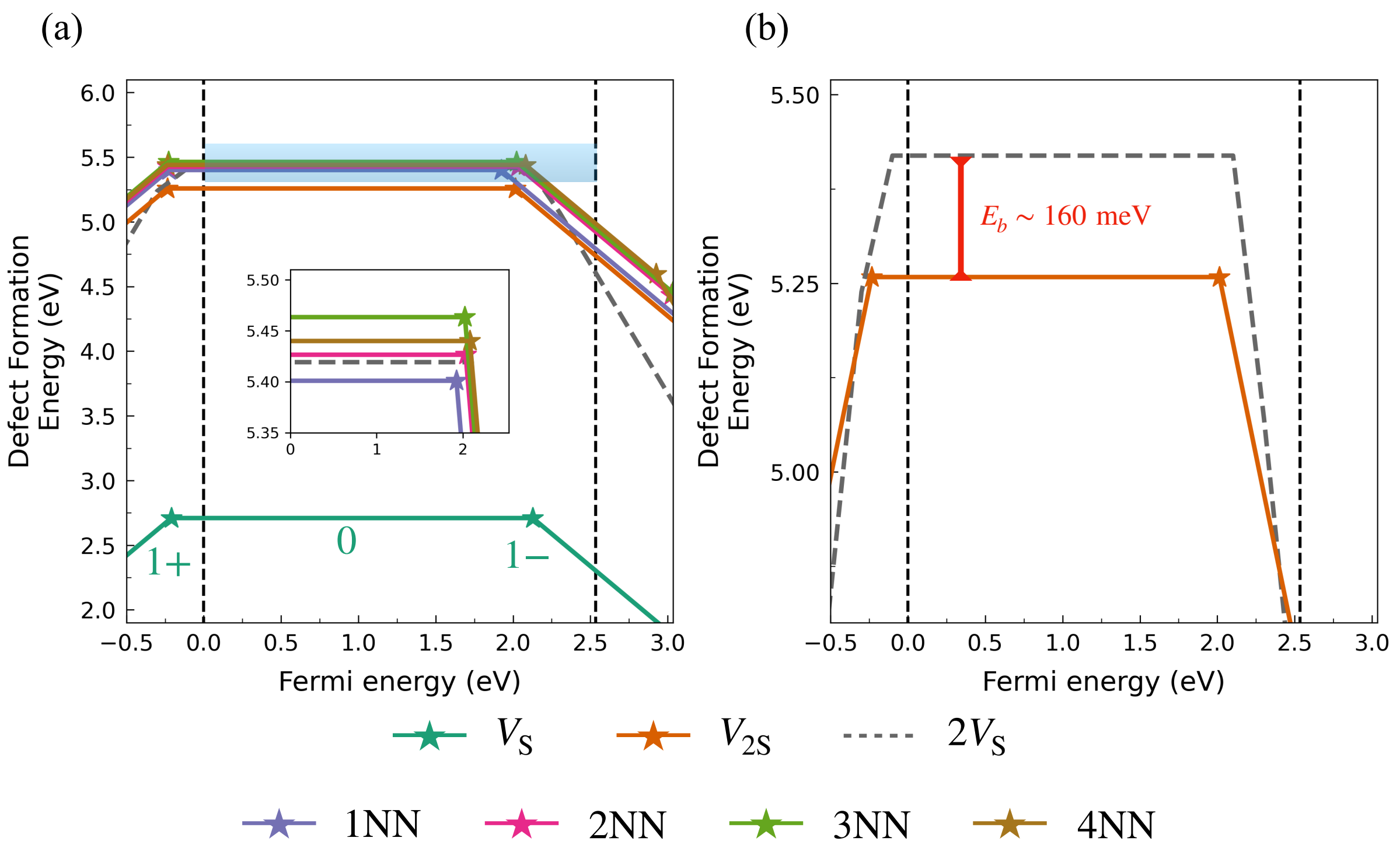

Figure S1: Formation energy of sulfur single vacancy V_S, divacancy V_{2S}, and nearest neighbor complexes 1NN to 4NN from charge state 2- to 2+ evaluated under sulfur poor condition (a). Gray dashed line indicating two infinitely separated vacancies $2V_S$ competing with the formation of vacancy paries. Inset shows zoomed-in energy range marked in the shaded area. (b): a binding energy around 160 meV is observed by the divacancy configuration at neutral charge state. Band edge positions are marked with vertical dashed lines. Fermi energy is referenced to the VBM.

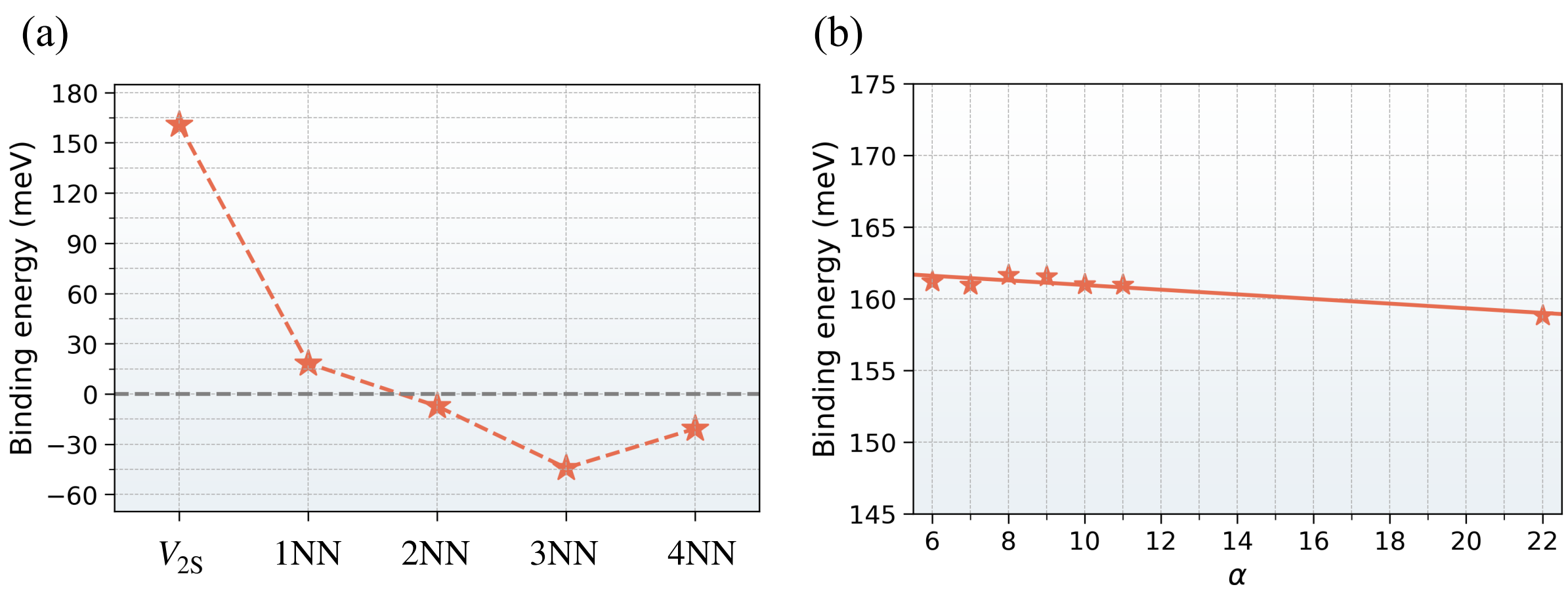

Figure S2: Computed binding energy, dependence on the amount of exact exchange (α) employed and the resulted equilibrium constant of divacancy formation. (a): binding energies for divacancy V_{2S} and nearest neighbor vacancy complexes 1NN through 4NN. (b): linear fit for the divacancy binding energy with different amount of exact exchange.

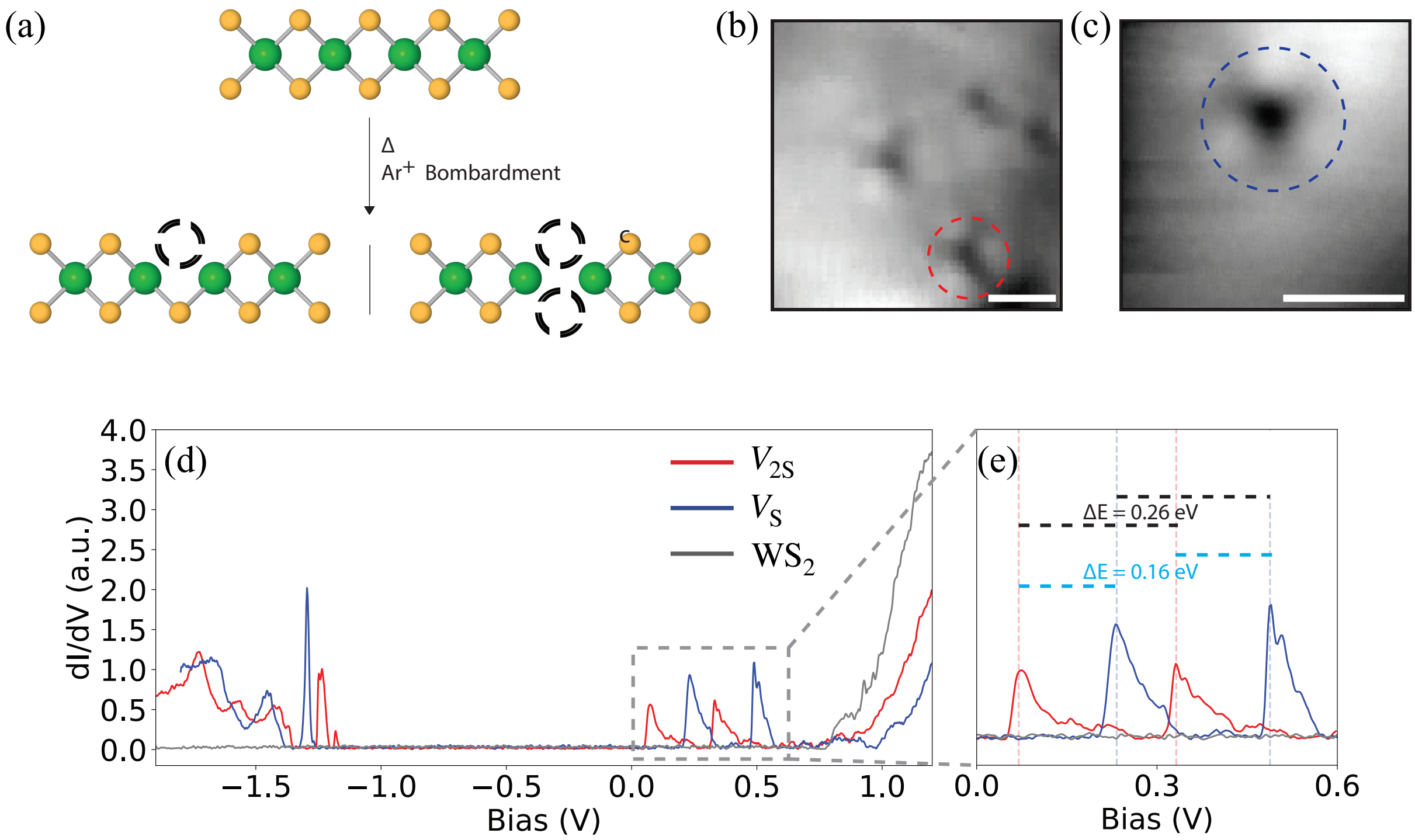

Figure S3: Divacancy scanning tunneling spectroscopy. (a): A schematic of our procedure to prepare defects within WS_2 is depicted. (b) and (c): Two defects are depicted that are indistinguishable in scanning tunneling microscopy (I_{tunnel} = 30 pA, V_{sample} = 1.2 V). Scale bars, 1 nm. (d), Scanning tunneling spectroscopy ($V_{modulation}$ = 5 mV) of each defect is shown (red, (b); blue, (c)), where an energetic shift of 0.16 eV is found ((d) and (e)) between both defects that otherwise exhibit matched spectroscopic signatures. We attribute this shift to the neutral divacancy V_{2S}^0 formation.

REFERENCES

[1] G. Kresse and J. Furthmüller, "Efficient iterative schemes for ab initio total-energy calculations using a plane-wave basis set," *Phys. Rev. B*, vol. 54, pp. 11169–11186, Oct 1996.

[2] G. Kresse and J. Furthmüller, "Efficiency of ab-initio total energy calculations for metals and semiconductors using a plane-wave basis set," *Computational Materials Science*, vol. 6, no. 1, pp. 15–50, 1996.

[3] P. E. Blöchl, "Projector augmented-wave method," *Phys. Rev. B*, vol. 50, pp. 17953–17979, Dec 1994.

[4] J. P. Perdew, M. Ernzerhof, and K. Burke, "Rationale for mixing exact exchange with density functional approximations," *J. Chem. Phys.*, vol. 105, no. 22, pp. 9982–9985, 1996.

[5] C. Adamo and V. Barone, "Toward reliable density functional methods without adjustable parameters: The pbe0 model," *J. Chem. Phys.*, vol. 110, no. 13, pp. 6158–6170, 1999.

[6] D. Broberg, B. Medasani, N. E. Zimmermann, G. Yu, A. Canning, M. Haranczyk, M. Asta, and G. Hautier, "Pycdt: A python toolkit for modeling point defects in semiconductors and insulators," *Computer Physics Communications*, vol. 226, pp. 165–179, 2018.

[7] H.-P. Komsa and A. Pasquarello, "Finite-size supercell correction for charged defects at surfaces and interfaces," *Phys. Rev. Lett.*, vol. 110, p. 095505, Feb 2013.

[8] H.-P. Komsa, N. Berseneva, A. V. Krasheninnikov, and R. M. Nieminen, "Charged point defects in the flatland: accurate formation energy calculations in two-dimensional materials," *Phys. Rev. X*, vol. 4, p. 031044, Sep 2014.

[9] M. F. Tabriz, B. Aradi, T. Frauenheim, and P. Deák, "Slabcc: Total energy correction code for charged periodic slab models," *Computer Physics Communications*, vol. 240, pp. 101–105, 2019.

[10] W. Chen, S. M. Griffin, G.-M. Rignanese, and G. Hautier, "Nonunique fraction of fock exchange for defects in two-dimensional materials," *Phys. Rev. B*, vol. 106, p. L161107, Oct 2022.
